# Front-line staff-led integrated care

**Published:** 2012-09-04

**Authors:** Gail Butt, Mel Krajden, Warren Hill

**Affiliations:** British Columbia Centre for Disease Control, University of British Columbia, Canada; British Columbia Centre for Disease Control, University of British Columbia, Canada; Children’s and Women’s Health Centre of British Columbia, Canada

**Keywords:** integrated care, nurse-led care, chronic illness, hepatitis C, complexity theory

## Abstract

**Purpose:**

To determine the number and type of providers and degree they work together to provide integrated chronic illness care for individuals with hepatitis C care in four small Canadian communities.

**Theory:**

A complexity theory-based model of front-line staff initiated partnerships for integrated care guided this descriptive comparative study.

**Methods:**

In 2008, front-line staff (nurses) in four hepatitis projects, initiated between 2001 and 2004, identified working relationships with multiple providers who were surveyed using measures of partnership structure and function. Data were analysed using descriptive statistics to determine partner characteristics, degree of integration and group functioning.

**Results and conclusions:**

The sample comprised 216 providers representing health, social and ancillary services. Sites operating for >5 years had the greatest number and diversity of partners. The sites were similar in the type of providers and quality of group functioning. Nurse-provider paired ratings for each site evidenced high agreement indicating relationship stability. The partners differed in mean depth of involvement (integration), and differences between expected minus observed involvement.

Nurse-initiated relationships with multiple providers in response to client needs results in service integration. Future studies should include input from care recipients to determine how well the provider mix meets their needs.

